# Contraceptive Risk Events among Family Planning Specialists: a Cross Sectional Study

**DOI:** 10.21203/rs.3.rs-4018351/v1

**Published:** 2024-03-08

**Authors:** Taylor N. Weckstein, Rebecca G. Simmons, Jami Baayd, Kathryn E. Fay

**Affiliations:** Harvard Medical School; University of Utah School of Medicine; University of Utah School of Medicine; Harvard Medical School

**Keywords:** contraception, contraceptive effectiveness, contraceptive failure, family planning clinicians, induced abortion, postcoital contraceptive, sexual health, unprotected intercourse

## Abstract

**Background:**

Proponents of abortion restriction cite advancements in contraceptive technology as a reason against the need for abortion care today, most recently through oral arguments in the Supreme Court of the United States case, *Dobbs v. Jackson Women’s Health*. However, consistent and correct use of contraception requires reproductive health literacy. Our objectives were to quantify contraceptive risk events and assess contraceptive history and preferences among a population well-equipped to evade contraceptive risks, family planning specialists following initiation of their medical training. “Risk events” are defined as reported episodes of contraceptive failure, emergency contraception use and/or unprotected or underprotected intercourse.

**Methods:**

This was a cross-sectional study among current members of a professional organization of family planning specialists. Inclusion criteria included: status as a current or retired clinician, consensual penile-vaginal intercourse since the start of medical training, and personal or partner capacity to become pregnant. Descriptive statistics were performed. This study was IRB exempt.

**Results:**

Among 229 respondents, 157 (69%) reported experiencing a contraceptive risk event since training. Twenty-nine (13%) respondents reported an occurrence within the last year. By category, 47% (108/229; 3 reported unknown) reported under- or unprotected intercourse, 35% (81/229) reported emergency contraception use, and 52% of participants (117/227; 2 unknown) reported known or suspected contraceptive failure. The mean number of contraceptive methods used was 3.7 (SD 1.7) out of the 13 methods listed. Almost all (97%) participants reported at least one method was not an acceptable option, with a mean of 5.6 (SD 2.7) of the 13 listed methods.

**Conclusions:**

The majority of family planning specialists have experienced contraceptive risk events during times of active pregnancy prevention since their medical training. Contraceptive method change is common and most respondents were limited in the number of methods that were personally acceptable to them. Dialogue idealizing the role of contraception in minimizing or eliminating abortion need is simplistic and inaccurately represents the lived realities of pregnancy-capable individuals and their partners, including among those with exceptional contraceptive literacy and access.

## Plain English Summary

Since *Dobbs v. Jackson*, the landmark Supreme Court of the United States case overturning the right to abortion, it is very important to better understand current birth control use and the risk of unwanted pregnancy. While birth control helps people to decide whether or not they become pregnant, birth control is not perfect. This study examined the limitations of current birth control, even when used by expert clinicians with special knowledge and access. We provided an online survey to doctors and advanced practice clinicians who specialize in birth control. We measured risk of unwanted pregnancy by asking about experiences with birth control failure, emergency contraceptive use (such as plan B), and unprotected sex since the start of medical training. We also asked about reasons for changing or avoiding certain birth control methods. Among 229 expert clinicians, we found that nearly 70% had experienced a risk of unwanted pregnancy since beginning their medical training. Birth control method change was common, and many reported that several options were unacceptable. Participants shared that they found methods difficult, unreliable, unpleasant, or had health conditions that limited the number of safe options available. Our findings suggest that, even among experts, everyone remains at risk of unwanted pregnancy. The study highlights the need for improved birth control options as well as access to safe and legal abortion.

## Background

Major advancements in contraceptive technology since the 1960s has been cited as a reason against the need for abortion care today, recently in the pivotal Supreme Court case, *Dobbs v. Jackson Women’s Health*. Specifically, oral arguments in the Dobbs case contended that “contraception is more accessible and affordable and available than it was at the time of *Roe* or *Casey*. It serves the same goal of allowing women to decide if, when, and how many children to have.”^1^ Justice Barrett also remarked that safe-haven laws further mitigate concerns regarding unwanted parenthood. Thus, authoritative sources have made a simplified conclusion that between contraception and adoption placement, the role of abortion is not as relevant as it was at the time of *Roe’s* passage.

Consistent and correct use of contraception requires access, health literacy, tolerance of side effects, and for some methods, a willing partner. Even among recent medical school graduates, however, contraceptive knowledge is low.^2^ Further, despite rigorous medical training, physicians report high rates of unprotected intercourse when not seeking conception and when partner sexually transmitted infection status is unknown.^3^ It follows that abortion is not uncommon (11.5%) among physicians measured over the life course.^4^ Thus, even among trained healthcare experts, let alone the general public, contraception does not eliminate the possibility of undesired pregnancy.

There are few populations more knowledgeable about contraceptive use and fertility than clinicians specializing in family planning. The purpose of this study was to assess the contracepting behaviors among this highly specialized group of individuals during their professional practice to assess whether their expertise was sufficient to nullify the risk of unwanted pregnancy. While unlikely, whether the lived experience of contemporary contraception use fully delivers on the point of deciding if, when, and how a pregnancy occurs, must be measured, starting with a population well-equipped to evade contraceptive risks.

## Methods

This was a cross-sectional study exploring contraceptive practices and risk of unwanted pregnancy among reproductive health experts during their professional careers. Participants were members of the Society of Family Planning, a professional reproductive health organization, including physicians, physician assistants, certified midwives, and nurse practitioners. Individuals were invited to participate through an email communication in the Society of Family Planning email listserv and a posting on an online research message board, available exclusively to members of the Society of Family Planning. There were two reminders to participate. Recruitment occurred between June 2022 to December 2022. Surveys were self-administered with data collection and management using REDCap electronic data capture tools hosted by Mass General Brigham Research Computing, Enterprise Research Infrastructure & Services (ERIS) group. REDCap (Research Electronic Data Capture) is a secure, web-based application designed to support data capture for research studies.^5^ The first page of the survey included a consent fact sheet; consent was implied by survey continuation. Inclusion criteria were 1) report of penile-vaginal intercourse since starting medical training; 2) personal or partner capacity to become pregnant since starting medical training; and 3) status as a current or retired clinician. This study consulted the CHERRIES (The Checklist for Reporting Results of Internet E-Surveys) reporting guidelines.^6^

Our primary outcome measured contraceptive risk events: times when participants or their partners were at potential risk of pregnancy when not seeking conception. We defined this measure through three questions: 1) How many different times have you or a sexual partner used emergency contraception, including oral medications and IUDs? 2) Have you had consensual penile-vaginal intercourse without using contraception (other than emergency contraception) or partial penile-vaginal intercourse (partial meaning starting intercourse without a condom or other contraceptive method, but using one before ejaculation, using the pullout method, etc.) when you or a sexual partner wanted to prevent pregnancy? and 3) Have you had consensual penile-vaginal intercourse and thought the contraception may have failed? We defined underprotected and unprotected intercourse using adapted items from Aiken and Trussell.^3^ Specifically, we measured underprotected intercourse to document the common practice of beginning intercourse without a form of contraception, given the risk of sperm exposure in pre-ejaculatory fluid, while also including withdrawal as a method of contraception.

We also included survey questions about participants’ contraceptive history. Participants reported all methods (n = 13) used personally or by their partner since the start of medical training and their reasons for discontinuing each method not currently being used. Participants were also asked about any method they would not want to use and their reasons for avoidance. The survey included write-in options for participants who indicated “other” as a reason for contraceptive method discontinuation and avoidance. These items were developed as adaptations from the National Survey for Family Growth, The Henry J. Kaiser Family Foundation, and Nelson *et al*.^7–9^ The survey concluded with demographic items using items adapted from Kaplowitz and Laroche.^10^ Item display order was not randomized or alternated; however, conditional display was utilized to supply additional questions only to participants who answered affirmatively to contraceptive risk events (to determine recency of event), method discontinuation items and method avoidance items (to identify reasons for discontinuation or avoidance for only and each method selected). No survey items required a response beyond the three initial screening items. Survey items were pilot testing among five medical professionals. At the end of the web-based survey, participants were invited to enter a drawing for a gift card via a separate survey link to preserve anonymity. Data storage was protected behind an institutional firewall.

We conducted descriptive statistical analyses to illustrate the prevalence of contraceptive risk events, method use, and reasons for method discontinuation or avoidance. This was a convenience sample; the sampling frame was determined by active registration as clinician with the Society of Family Planning. Qualitative responses to the write-in questions were thematically coded and compiled. All data analyses were conducted in STATA (StatCorp, 2019, College Station, TX). The study was reviewed by the Mass General Brigham Institutional Review Board and deemed exempt (2022P001454)

## Results

**Figure 1:**
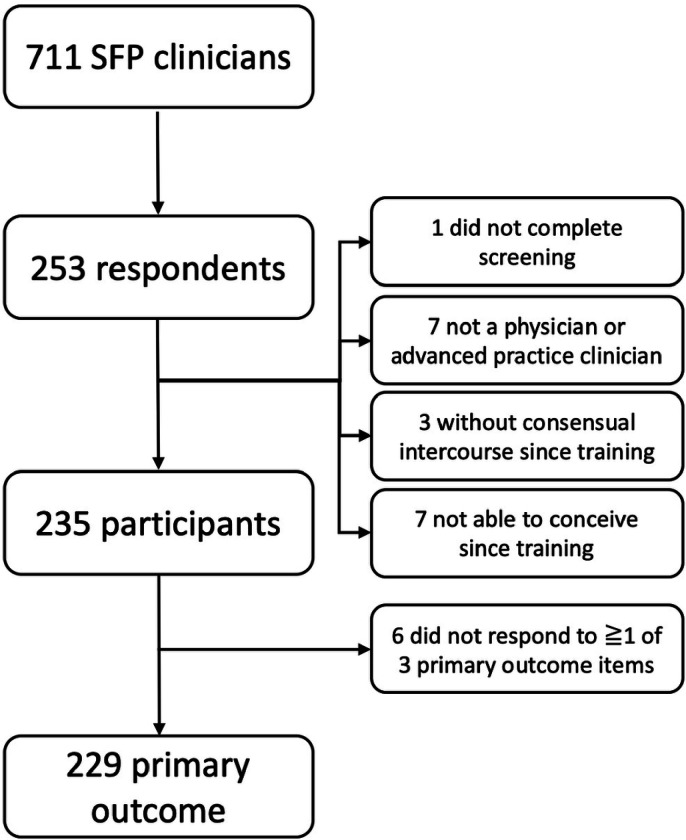
Study Flow. Sample size based on response rate, inclusion criteria, and completion of primary outcome items.

Of 711 currently registered clinicians, 253 (36% click rate) opened the survey invitation, and 229 (91% completion; 32% total sample frame) answered at least one of the three primary outcome items (Fig. 1). Participants trained across 35 states and Washington, D.C. The majority of respondents identified as women (95%), and the majority of respondents reported completing an MD degree (85%). Nearly half of participants (49%) responded that it had been one to two decades since they completed their training. A full summary of demographic data is included in Table 1.

**Figure 2:**
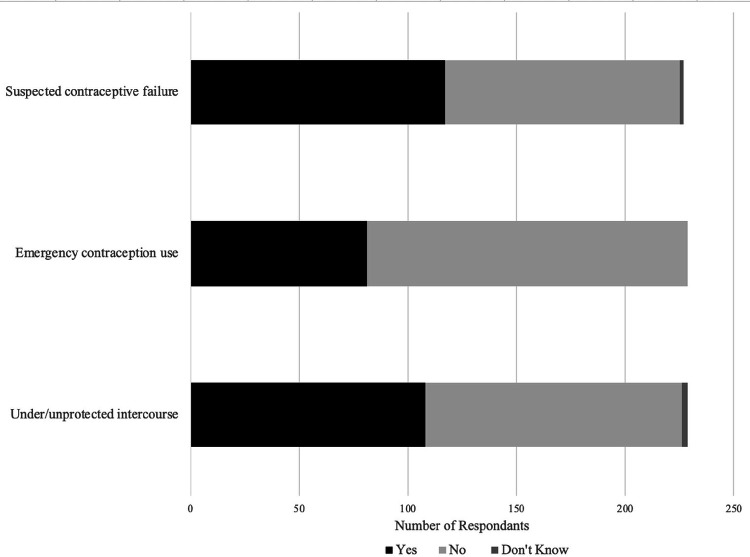
Primary Outcome: Contraceptive Risk Events. Number of respondents reporting a contraceptive risk event since the start of their medical training. Events reported as a risk incurred by the dyadic unit, the respondent and sexual partner.

Overall, 69% (n = 157) of respondents reported at least one contraceptive risk event during a time when pregnancy prevention was undesired (Fig. 2). Four individuals reported they did not know if a risk event had occurred. Thirteen percent (29/229) of all participants reported that the risk event occurred within the past year; all but one of these individuals reported being more than six years into their career. By risk event, 47% (108/229; 3 reported unknown) reported under- or unprotected intercourse, 35% (81/229) reported emergency contraception use, and 52% of participants (117/227; 2 reported unknown) reported known or suspected contraceptive failure since their training. Among emergency contraception users, seventeen individuals reported using emergency contraception more than three times, with some individuals (3/81) reporting use more than ten times.

**Figure 3:**
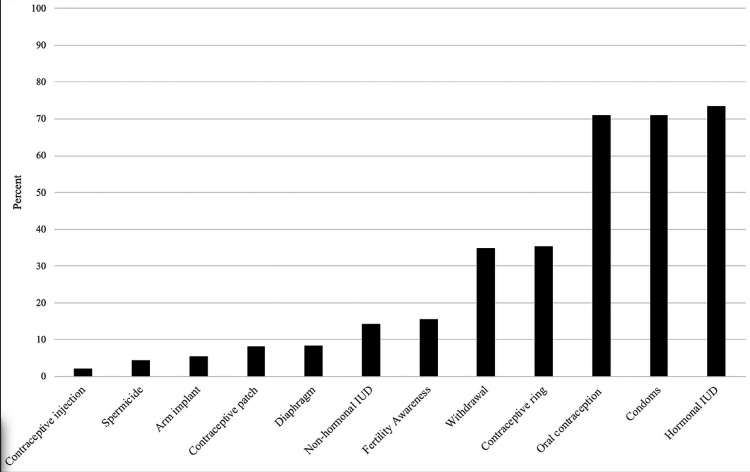
^1^Methods used. Percentage of respondents reporting personal or partner use of a contraceptive method since the start of their training. ^1^While emergency contraception (EC) can be an individual’s primary method for pregnancy prevention, we used EC as an indicator of risk and omitted it from this Figure. That is, we conceptualized EC use as a behavior in response to a contraceptive risk event.

The most common contraceptive methods participants reported using since beginning their medical training were hormonal IUDs, condoms and oral contraception; for each method, more than 70% of respondents reported current or prior use. Contraceptive injection, spermicide, arm implant, contraceptive patch, and diaphragm were uncommon methods among family planning clinicians, with less than 10% of respondents reporting prior use per method (Fig. 3).

**Figure 4:**
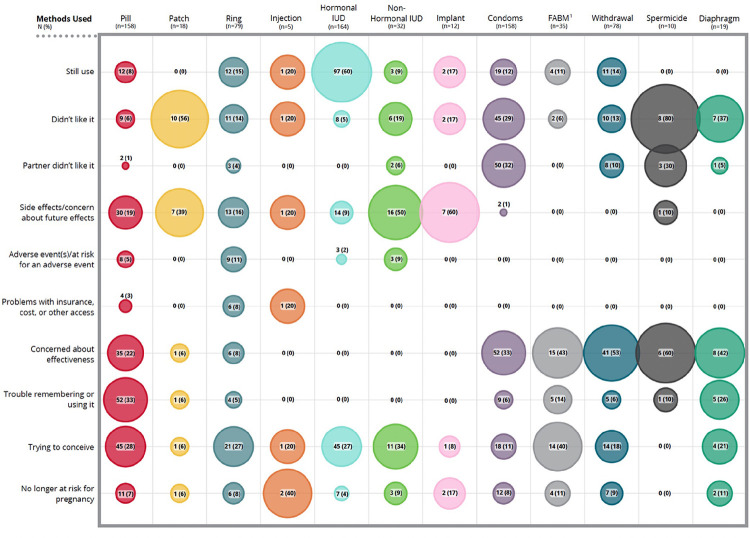
Reasons for method discontinuation. Contributing factors for method discontinuation among prior methods used by respondents and/or their sexual partner.

**Figure 5:**
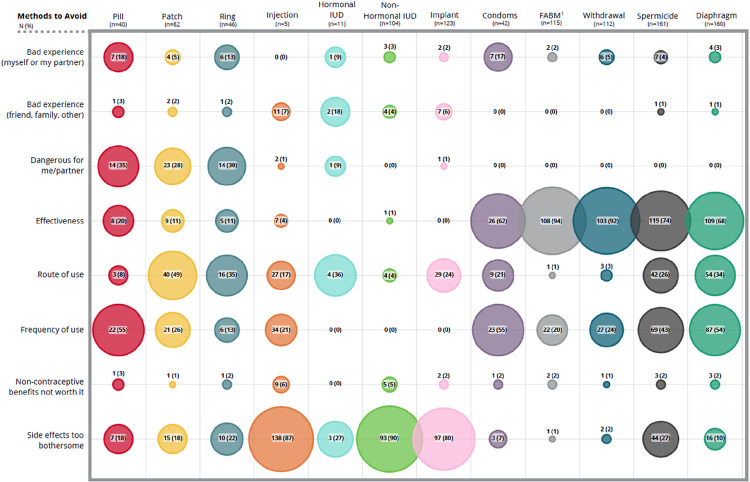
Reasons for method avoidance. Contributing factors for method avoidance by respondents and/or their sexual partner. Respondents checked all that applied.

The mean number of contraceptive methods used since medical training was 3.7 (SD 1.7), including emergency contraception. Almost all (215/222, 97%) participants reported they would personally avoid at least one type of contraceptive method. On average, participants reported that 5.6 (SD 2.7) of the 13 methods available would be unacceptable to them for personal use. Figures 4 and 5 shows reasons participants reported deciding to discontinue or avoid various methods. Table 2 displays write-in “other” reasons respondents chose to discontinue or avoid particular methods [see Additional file 1].

## Discussion

Approximately 7 in 10 family planning specialists reported a contraceptive risk event during their professional careers when pregnancy prevention was desired. While most participants were over a decade into their careers, 29 (13%) reported a risk event within the past year. These data show that even in the context of significant knowledge and high uptake of the most effective methods, risk of unintended pregnancy persists, underscoring the need for robust abortion access.

Our findings parallel metrics of similar contraceptive risks events in the general public. In an analysis of a national population of reproductive-aged women in 2015, 23% reported prior emergency contraception use, less than in our sample (35%),^11^ which may be explained by improved access to, knowledge of, and comfort with reporting use of this contraceptive method among family planning specialists. This reported higher use among our study population may also be impacted by the measure of ever use since training (including older individuals, not just reproductive aged) and ongoing increases in use since 2015 facilitated by lower costs and easier acquisition of emergency contraception. Participants’ report of under- or unprotected intercourse was similar to findings from a survey administered in 2014 to family planning specialists using the same definition: 76% lifetime risk and 7% past-year risk. Our ever-risk is likely lower because our query was limited to time since training commenced; our past-year risk of 13% may be accounted for by omission of withdrawal from the comparative study’s figure.^3^ Regardless, among the family planning clinician population, contraceptive risks have been, and continue to be, part of the lived experience after the initiation of medical training. We measured perceived failure rather than pregnancy incidence. In a population of users highly trained to identify failure like incorrect or inconsistent use or device expulsion, capturing the potential for pregnancy may better address our research question than the overestimated performance deduced from clinically recognizable pregnancy used to calculate Pearl indices. Consequently, we refrain from situating our final metric of contraceptive failure in the context of the general typical use effectiveness measuring pregnancy incidence.

The most common methods used among both participants and the US population include oral contraception, external condoms, and intrauterine devices (IUDs), with a higher rate of IUD use among our participants compared to the general US population.^12^ The hormonal IUD was the most common method still being used with the highest rate of discontinuation for planned conception. Individuals remain at risk of pregnancy, unsurprisingly, even with perfect use of contraception; multiple participants described experiences of IUD failures. However, it is unrealistic and unforgiving to expect that anyone—including reproductive health experts—will have perfect contraceptive use at each sexual encounter for a multitude of reasons, including the shortcomings of currently available contraceptive methods. Adverse effects and problems with access were infrequently reported reasons for discontinuation of a method in this cohort. However, side effects were a common reason for method avoidance, particularly for the injection, nonhormonal IUD and implant. Prior study has found that among first time contraceptive users, nearly half were worried about side effects before starting contraception; however, the degree to which these concerns have contributed to method avoidance among the general public is not clear. Given the unique expertise of family planning providers, extensive knowledge around potential side effects across methods likely contributed to informed decision-making and method avoidance. Participants’ report of side effects had overlap with other studies including bleeding and interference with sexual pleasure; although, based on write-in responses, weight and mood concerns were underrepresented in this population.^8^

Participants also reported development of contraindications. In other studies, up to one third of individuals using combined oral contraceptives reported a relative or absolute contraindication to use, due to medical comorbidities.^13,14^ The high prevalence of these comorbidities may markedly limit the number of contraceptive options safely available to many pregnancy-capable individuals. Notably, participants echoed the sentiments of many other contraceptive users in emphasizing the importance of control over the method – rather than reliance on a partner for use or a clinician for initiation or discontinuation.^15–17^ As is the case in all populations, there are a diverse set of factors contributing to the (un)desirability of a contraceptive method, again highlighting that effectiveness is not the only metric influencing contraceptive decision-making. This is consistent with other work demonstrating that the contraceptive decision making process is often a dynamic and nuanced process that changes over the course of decades.^18^ Contraceptive decision-making changes with changing bodies, belief systems, environments and relationships.^19^

In examining a population with a unique knowledge base and likely excellent access to contraception, including long-acting reversible methods, contraceptive risk events are common over the course of individuals’ professional lives, as is method discontinuation (for reasons other than conception) and method avoidance. These findings normalize contraceptive risk behaviors, emphasize that “typical use” describes use among all contraceptive users, and highlights the narrow range of contraceptive choice when accounting for method contraindications, performance features, and evolving user preferences. These findings work to dismantle the idea of an ideal contraceptive method or contraceptive user in an era characterized by intolerance of undesired pregnancy and loss of abortion access. Such considerations factor into clinical care, by, for example, reducing “otherization” in contraceptive counseling, building empathy for contraceptive dissatisfaction, and expanding the image of potential abortion beneficiaries to everyone. More tangibly, this translates to provision of universal guidance and access to emergency contraception, counseling on the reality of contraceptive switching and discontinuation for many users, and consideration of the inclusion of abortion counseling with contraceptive counseling.^20^

These data have implications for the contemporary social and environmental factors affecting sexual and reproductive health by highlighting contraceptive shortcomings and events representing potential abortion need. Further exploration of contraceptive dissatisfaction may facilitate public understanding of the limitations of contraceptive technology and the demands put on pregnancy-capable people in navigating method use. These findings also emphasize the need for expansion of contraceptive options with critical research focused on development of novel agents and delivery systems, including male hormonal contraceptive methods.^21^

The strengths of this study include its unique insight into contraceptive risk behavior and contraceptive choices among family planning specialists using quantitative and qualitative input. These are salient data for generating a response to current questions around the role of contraception, particularly as it pertains to abortion need. Our study is limited by a design that did not allow for a comparison between contraceptive risk event and method at the time of event. However, the focus of this study was on the prevalence of risk in a population with access to and knowledge about all contraceptive options; the relevance of method data was intentionally focused on exploring imperfections of current technology. Our survey did not include specific questions about how a contraceptive method impacted sexual pleasure or bodily autonomy, themes subsequently noted in the qualitative responses that should be included in future work. Finally, our response rate, while consistent with or better than most online surveys, may be subject to non-response bias, including the possibility of preferential response among those with a specific interest in sharing their contraceptive risk histories.^22^ While demographics of the Society of Family Planning membership are not publicly available data, the geographic diversity of this sample is similar to those in the member directory providing support of generalizability along one dimension.

## Conclusions

Family planning specialists report contraceptive risk events while actively avoiding pregnancy; thus, optimization of the role of contraception with education and access do not generate immunity to abortion need. Advances in contraceptive method diversity and technology should be celebrated, as should contraceptive uptake that meets the needs of its user. However, dialogue focusing on the role of contraception in minimizing or eliminating abortion need perpetuates stigma around abortion and does not accurately represent individuals’ lived experiences, including those with significant educational and social privilege like family planning specialists.^23^ Further, like all contraceptive users, those with specialized knowledge of contraception also use individualized algebra to determine method goodness of fit, not limited to considerations of efficacy. Contraceptive preferences and method avoidance are driven by practical and important concerns, like side effects and ease of use, that greatly reduces the menu of options available to the contemporary contraceptive user. Contraception has not and never will eliminate the need for abortion, even among individuals with considerable personal interest and professional training in contraception. In the wake of significant losses in abortion protection, the expansion of contraception options and abortion access, together, should be celebrated in the effort to support reproductive liberty.

## Figures and Tables

**Table 1:** Demographic Information

	N(%)^1^ (219)
Gender identity^2^	
Woman/female/feminine	208 (95)
Man/male/masculine	11 (5)
Age	
20–29 years	6 (3)
30–39 years	114 (52)
40–49 years	70 (32)
50–59 years	21 (10)
60–69 years	6 (3)
70 years or more	2 (1)
Credentials	
CNM	9 (4)
DO	9 (4)
PA	1 (0.5)
NP/DNP	15 (7)
MD	185 (85)
Years since training	
< 12 months	1 (0.5)
1–2 years	2 (1)
3–5 years	6 (3)
6–10 years	53 (24)
11–20 years	107 (49)
> 20 years	50 (23)
Region^3^	
Northeast	89 (43)
Midwest	33 (16)
South	35 (17)
West	48 (23)

1 May not sum to 100 due to rounding.

2 Respondents were also given the option of transgender woman, transgender man, gender expansive, prefer to self-describe, and prefer not to answer; no respondents selected these options.

3 n=205

## Data Availability

The datasets generated during the current study are available from the corresponding author on reasonable request.

## Abbreviations

IRB: Institutional Review Board
REDCap: Research Electronic Data Capture
ERIS: Enterprise Research Infrastructure & Services
CHERRIES: The Checklist for Reporting Results of Internet E-Surveys
IUD: Intrauterine Device
SD: Standard Deviation
